# Successful treatment of fulminant myocarditis with intra-aortic balloon pump counterpulsation combined with immunoglobulin and glucocorticoid in a young male adult

**DOI:** 10.3389/fcvm.2022.905189

**Published:** 2022-07-22

**Authors:** Huanhuan Li, Lun Li

**Affiliations:** Department of Cardiology, Wuhan Fourth Hospital, Wuhan, China

**Keywords:** fulminant myocarditis, intra-aortic balloon counterpulsation pump, immunoglobulin, glucocorticoid, cardiogenic shock, multiple organ failure, case report

## Abstract

**Background:**

Fulminant myocarditis (FM) is a serious non-specific inflammatory disease of the myocardium. FM tends to occur in adolescents and the course of the disease progresses rapidly. It is prone to cardiogenic shock (CGS) and multiple organ failure (MOF) with high mortality. We report a case of FM with CGS and MOF in a young male who was successfully treated with intra-aortic balloon pump counterpulsation (IABP) combined with intravenous immunoglobulin (IVIG) and glucocorticoid (GC).

**Case summary:**

A 21-year-old previously healthy man presented with fever, headache, and chest tightness. He came to the hospital for emergency treatment. The laboratory data showed that the levels of serum cardiac troponin I (cTnI), N-terminal B-type natriuretic peptide (NT-proBNP), myocardial zymogram, and neutrophils increased. Echocardiography showed pericardial effusion and decreased left ventricular systolic function. ECG showed diffuse ST-segment elevation. He was clinically diagnosed with FM and admitted to the intensive care unit for treatment. Within 48 h of admission, the clinical course of the patient deteriorated rapidly, with CGS accompanied by MOF, high atrioventricular block (AVB), and ventricular tachycardia (VT). After using mechanical circulatory support (MCS) therapy with IABP, IVIG, GC, continuous renal replacement therapy (CRRT), and mechanical ventilation complicated with a temporary cardiac pacemaker, he recovered normal cardiac function. He made a full recovery and was discharged home on day 21.

**Discussion:**

For patients with FM, early diagnosis, close monitoring, timely use of MCS devices, and active comprehensive treatment are very important. MCS devices such as IABP can become lifesaving tools for the treatment of FM.

## Introduction

Fulminant myocarditis (FM) is a sudden and life-threatening myocardial disease, which is characterized by atypical clinical symptoms, rapid disease progression, and a high risk of death without timely treatment (1–3). Viral infection is the most common cause of FM (4). When FM occurs, the virus causes direct damage to the myocardium and a secondary immune response causes indirect damage to the myocardium. These cause rapid and serious damage to the heart, resulting in the rapid decline in cardiac function and serious hemodynamic disorders (5, 6). Mechanical circulatory support (MCS) devices are recommended for patients with FM with poor response to drug therapy (3, 7). We describe a young male FM case of cardiogenic shock complicated with multiple organ failure (MOF), which was successfully treated with intra-aortic balloon pump counterpulsation (IABP) combined with intravenous immunoglobulin (IVIG) and glucocorticoid (GC).

## Case presentation

On 17 August 2018, a previously healthy 21-year-old man living in Wuhan developed fever, headache, and chest tightness after catching a cold, with a maximum temperature of 39.0°C. He also developed symptoms of dry cough, nausea and vomiting. He did not suffer from syncope, abdominal pain, diarrhea, jaundice, black stool, frequent urination, urgent urination, pain, and other discomforts. He went to the community hospital to see a doctor and was treated with mezlocillin, sulbactam, and diclofenac sodium. The symptoms of headache and chest tightness could not be relieved. At the same time, he had dyspnea and decreased activity. For further treatment, he came to Wuhan Fourth Hospital at 5 p.m. on 21 August 2018. He had no history of heart disease, no family history of heart disease, and no history of food or drug allergies. He did not smoke or drink alcohol and had not undergone any major surgery, trauma, blood transfusion, or intravenous drug abuse before onset.

**FIGURE 1:**
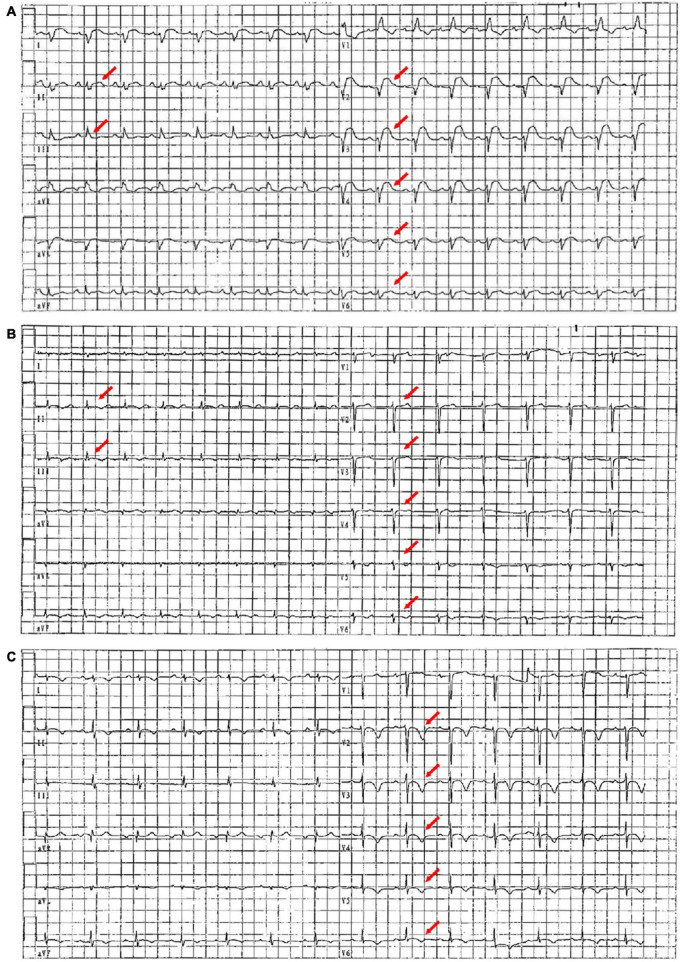
ECG images on the first day, the seventh day, and the 20th day of admission. **(A)** On the first day of admission, ECG showed sinus rhythm, right bundle branch block, and diffuse ST-segment elevation (red arrow). **(B)** On the seventh day of admission, ECG showed sinus rhythm and diffuse ST-segment elevation that improved significantly (red arrow). **(C)** On the 20th day of admission, ECG showed sinus rhythm with extensive anterior wall T-wave inversion (red arrow).

**FIGURE 2:**
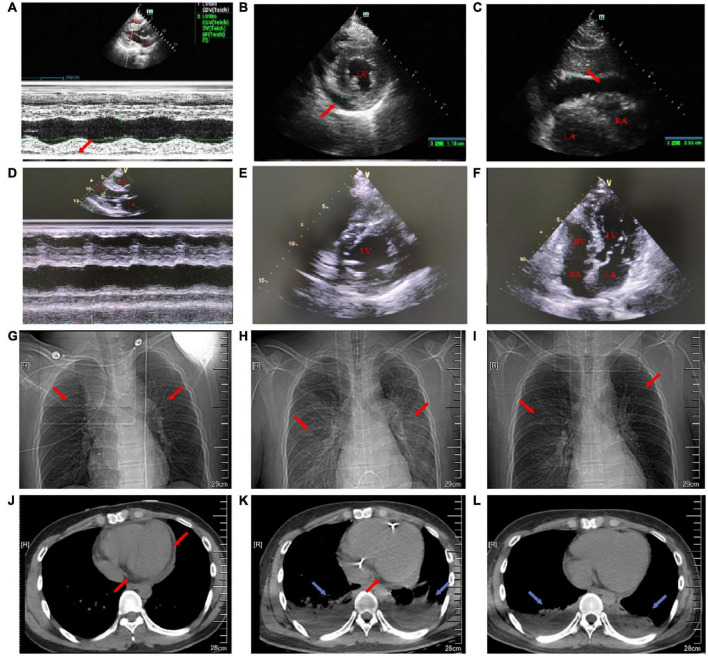
Transthoracic echocardiography on the first day and the 18th day of admission. Chest X-ray and chest CT images on the first, eighth, and 15th day of admission. **(A)** On the first day of admission, the maximum anterior–posterior diameter of the posterior dark area of the left ventricular posterior wall on the long-axis view of transthoracic echocardiography was 0.8 cm (red arrow). **(B)** On the first day of admission, the maximum anterior–posterior diameter of the dark area of the lower wall of the left ventricle on the short-axis view of transthoracic echocardiography was 1.2 cm (red arrow). **(C)** On the first day of admission, transthoracic echocardiography showed that the anterior–posterior diameter of the dark area under the xiphoid process was 2.5 cm (red arrow). **(D)** On the 18th day after admission, the pericardial effusion on the long-axis view of transthoracic echocardiography was less than that before. **(E)** On the 18th day after admission, the pericardial effusion on the short-axis view of transthoracic echocardiography was less than that before. **(F)** On the 18th day of admission, transthoracic echocardiography four-chamber view. **(G)** On the first day of admission, a chest X-ray showed a few fibrous foci in both the lungs (red arrow). **(H)** On the eighth day of admission, the chest X-ray showed inflammation in the upper lobe of the left lung and the middle lobe of the right lung (red arrow). **(I)** On the 15th day of admission, the chest X-ray showed a little inflammation of the left upper lung (red arrow). **(J)** On the first day of admission, the cross-section of the chest CT soft-tissue window showed that the pericardial fat space was blurred and there was a large amount of pericardial effusion (red arrow). **(K)** On the eighth day of admission, the cross-section of the chest CT soft-tissue window showed bilateral pleural effusion with bilateral lower lung insufficiency (blue arrow) and a small amount of pericardial effusion (red arrow). **(L)** On the 15th day of admission, the cross-section of the chest CT soft-tissue window showed bilateral pleural effusion and bilateral lower lung insufficiency (blue arrow).

In the emergency department, his vital signs and physical examination were as follows: body temperature (T) 38.4°C, respiratory rate (RR) 23 beats/min (bpm), blood pressure (BP) 106/72 mm Hg, pulse (P) 98 bpm, oxygen saturation (SpO_2_) 98% (indoor air), pupil diameter 2 mm, and sensitive bilateral light reflex. No swelling of superficial lymph nodes is found in the whole body. The breath sounds of both the lungs were thick during auscultation and no dry and wet rales were heard. The heart rate (HR) was 98 bpm, the heart sound was low and dull, and there was no murmur in each valve area. The abdomen was flat and soft, the Murphy sign was negative, the liver, spleen, and subcostal were not reached, there was no edema in both the lower limbs, and the pathological sign was negative. Admission laboratory tests showed (Supplementary Table 1): leukocyte count 6.75 × 10^Λ^ 9/L, neutrophils 68.2%, lymphocytes 18.7%, eosinophils 0.3%, amylase 24 U/l, total bilirubin 27.6 μmol/l, direct bilirubin 13.3 μmol/l, blood potassium 3.5 mmol/l, creatinine 107.5 μmol/l, random blood glucose 7.51 mmol/l, cardiac troponin I (cTnI) 38.678 ng/ml, N-terminal B-type natriuretic peptide (NT-proBNP) 8,480 ng/ml, creatine kinase (CK) 1,203 U/l, creatine kinase-MB (CK-MB) 55 U/l, lactate dehydrogenase (LDH) 406 U/l, alanine aminotransferase (ALT) 21 U/l, aspartate aminotransferase (AST) 114 U/l, and procalcitonin (PCT) 0.47 ng/ml. ECG showed: sinus rhythm, right bundle branch block, and ST-segment elevation in leads II, III, aV_*F*_, and V_2_–V_6_ (diffuse) (Figure 1A). Transthoracic echocardiography showed pericardial effusion [the maximum anterior–posterior diameter of the posterior dark area of the left ventricular posterior wall was 0.8 cm (Figure 2A), the maximum anterior–posterior diameter of the dark area of the left ventricular inferior wall was 1.2 cm (Figure 2B), and the anterior–posterior diameter of the dark area of the xiphoid process was 2.5 cm (Figure 2C)] and the left ventricular systolic function decreased [left ventricular ejection fraction (LVEF) = 42%]. Brain CT showed no abnormality. Chest CT showed a few fibrous foci in both the lungs (Figure 2G). Massive pericardial effusion and blurred pericardial fat space are also present, considering the possibility of inflammatory lesions (Figure 2J). Abdominal CT showed intrahepatic bile duct calculi.

As a result of these clinical findings, he was suspected of being diagnosed with FM and was admitted to the intensive care unit. Anti-infective (piperacillin and sulbactam) and antiviral (astragalus and oseltamivir) treatments were given; vitamin C and coenzyme Q10 were started to be taken. Then, we screened the viruses that may cause myocarditis, namely, coxsackievirus (CV) B3, CV B5, enterovirus, and cytomegalovirus, which were negative. The antimyocardial virus antibody test was negative. Other viral screening tests, such as hepatitis antibody, HIV antibody, and syphilis antibody, were negative (Supplementary Table 1).

**FIGURE 3:**
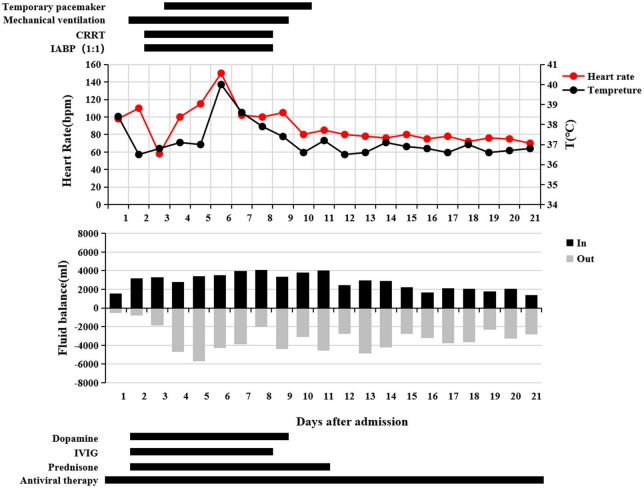
Clinical course of the patient during 21 days of hospitalization. Heart rate and body temperature changes. Daily liquid inflow and outflow. Use mechanical support therapy and medication.

His clinical course was as follows (Figure 3). His body temperature gradually decreased 24 h after admission, but he still had headache, nausea, and vomiting. The output was significantly less than the input. The vital signs monitoring showed that: temperature 36.5°C, RR 24 bpm, BP 90/60 mm Hg, HR 110 bpm, and SpO_2_ 92% (nasal catheter oxygen inhalation). The results of myocardial zymogram and cardiac injury markers increased further: cTnI 36.2786 ng/ml, NT-proBNP 13,427 ng/ml, CK 1,694 U/l, CK-MB 90 U/l, LDH 491 U/l, ALT 47 U/l, AST 154 U/l, creatinine 102.3 μmol/l, total bilirubin 25 μmol/l, direct bilirubin 11.6 μmol/l, PCT 0.6 mg/dl, and arterial blood gas [pH 7.46, partial pressure of arterial carbon dioxide (PaCO_2_) 29 mm Hg, partial pressure of arterial oxygen (PaO_2_) 268 mm Hg, bicarbonate (HCO_3_^–^) 20.6 mmol/l, alkali residue (BE^–^) 2.2, and lactic acid (Lac) 2.1 mmol/l]. Combined with laboratory examination, FM combined with acute left heart failure and upper respiratory tract infection was considered. Non-invasive ventilator-assisted respiration (continuous positive airway pressure under 12 cm H_2_O to maintain adequate tissue oxygenation) was given and circulating agonists (dopamine 7.5 μg/kg/min) were added to maintain blood pressure. At the same time, IVIG (40 g/day in the first 2 days and gradually decrease in the next few days) and intravenous GC (200 mg/day of methylprednisolone in the first 3 days and gradually decreasing in the next few days) treatment started. Furosemide was injected intravenously to optimize intravascular volume.

On the 42nd h of admission, he developed irritability, panting, sitting up, and breathing and the output was still less than the intake. Physical examination suggested: temperature 36.8°C, RR 36 bpm, BP 90/42 mm Hg (maintained by 10 μg/kg/min of dopamine), HR 118 bpm, SpO_2_ 90% (non-invasive ventilator-assisted respiration), low respiratory sounds in both the lungs, and audible moist rales. Laboratory tests became worse: NT-proBNP 16,163 ng/ml, CK 1,809 U/l, CK-MB 110 U/l, LDH 1,469 U/l, ALT 47 U/l, AST 154 U/l, and the level of anaerobic metabolism in arterial blood is very high (pH 7.20, PaCO_2_ 26 mm Hg, PaO_2_ 73 mm Hg, HCO_3_^–^ 10.2 mmol/l, BE^–^ 13.6, and Lac 10.9 mmol/l). Echocardiography showed pericardial effusion (small to medium volume, the maximum anterior–posterior diameter of the dark area of the lower wall of the left ventricle was 2.0 cm, and the anterior–posterior diameter of the dark area of the inferior xiphoid process was 1.0 cm) and the left ventricular systolic function decreased further (LVEF = 38%). Color Doppler ultrasound of the thoracic cavity showed bilateral pleural effusion (5.9 cm on the left and 6.8 cm on the right). Surgical pericardium and pleural puncture were not performed because there was no safe puncture space. The patient was considered to have CGS combined with MOF and had a poor response to vasoactive drugs. After obtaining informed consent, we immediately gave IABP (counterpulsation pressure 110 mm Hg, counterpulsation ratio 1:1), continuous renal replacement therapy (CRRT), and endotracheal intubation with ventilator-assisted respiration (continuous positive airway pressure under 15 cm H_2_O to maintain sufficient tissue oxygenation) and then the upper gastric tube was given to ensure intestinal nutrition. He developed Adams–Stokes syndrome during CRRT and developed a third-degree atrioventricular block (AVB) after rescue. We gave him temporary pacemaker implantation. Continuous administration of heparin through an infusion pump maintained an activated partial thrombin time of 70–100 s to prevent left ventricular thrombosis. Intermittent transfusion ensured that a hemoglobin level was greater than 90 g/l.

**FIGURE 4:**
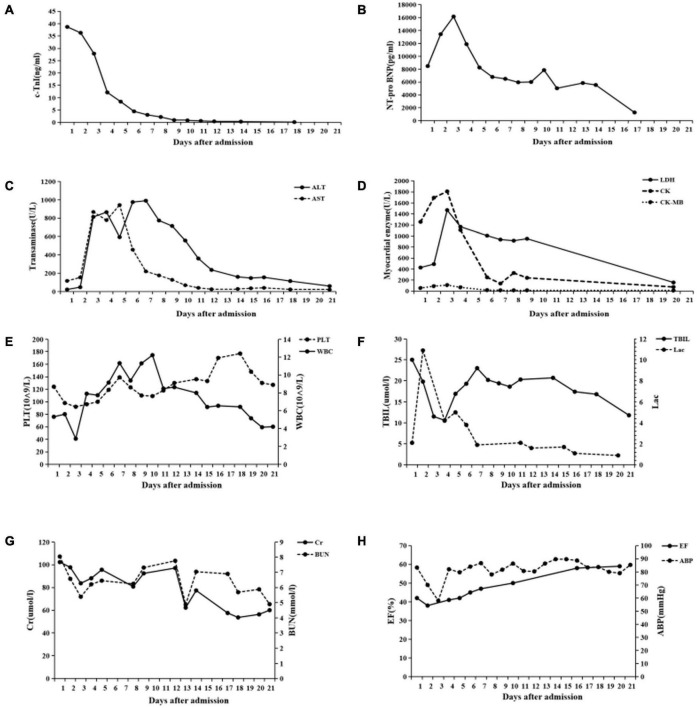
Changes of various indexes during 21 days of hospitalization. **(A)** cTnI. **(B)** NT-pro BNP. **(C)** ALT and AST. **(D)** Myocardial zymogram. **(E)** Platelet (PLT) and leukocyte count (WBC). **(F)** Total bilirubin (TBIL) and arterial blood gas lactate (Lac) levels. **(G)** Creatinine (Cr) and blood urea nitrogen (BUN). **(H)** Ejection fraction (EF) and average blood pressure (ABP) in transthoracic echocardiography.

The subsequent days of treatment were maintained using a combination of IABP, CRRT, mechanical ventilation, a temporary pacemaker, and drug support. The patient’s body temperature was normal, the urine output also continued to increase, and the water balance gradually tended to be negative (Figure 3). Cardiac injury markers such as cTnI (Figure 4A), NT-proBNP (Figure 4B), and myocardial zymogram tended to improve (Figures 4C,D), leukocyte count (Figure 4E), liver function (Figure 4F), and renal function (Figure 4G). Echocardiography indicates gradual improvement of LVEF (Figure 4H) and the level of anaerobic metabolism in arterial blood decreased (pH 7.40, PaCO_2_ 30 mm Hg, PaO_2_ 77 mm Hg, HCO_3_^–^ 18.6 mmol/l, BE^–^ 5.4, and Lac 5.0 mmol/l).

On the sixth day of admission, the patient had a fever again, with a temperature of 40.0°C. Three cases of ventricular tachycardia (VT) and ventricular fibrillation were corrected by electrocardiography and amiodarone.

On the seventh day of admission, the patient’s body temperature decreased and the level of anaerobic metabolism in arterial blood further decreased (pH 7.43, PaCO_2_ 33 mm Hg, PaO_2_ 90 mm Hg, HCO_3_^–^ 21.9 mmol/l, BE^–^ 1.9, and Lac 1.9 mmol/l), suggesting that organ perfusion was improved and relevant laboratory indexes tended to be improved (Figure 4). ECG showed that sinus rhythm and diffuse ST-segment elevation were significantly improved (Figure 1B). Echocardiography showed that the pericardial effusion decreased gradually (less to medium, the maximum anterior–posterior diameter of the dark area of the lower wall of the left ventricle was 0.8 cm) and the left ventricular systolic function was higher than that of front (LVEF = 47%). Color Doppler ultrasound of the thoracic cavity showed bilateral pleural effusion (2.6 cm on the left and 4.3 cm on the right). Brain CT showed no abnormality. Chest CT showed inflammation in the upper lobe of the left lung and the middle lobe of the right lung (Figure 2H), bilateral pleural effusion and bilateral lower lung insufflation (Figure 2K), and a small amount of pericardial effusion. Abdominal CT showed changes in peritoneal exudation and pelvic effusion. Considering the reduced demand for mechanical support, they stopped CRRT support.

On the ninth day of admission, IABP was pulled out, endotracheal intubation was pulled out, and ventilator treatment was stopped. The patient had no wheezing, dyspnea, and chest tightness. Physical examination showed: temperature 37.4°C, RR 19 bpm, BP 95/52 mm Hg, HR 105 bpm, SpO_2_ 97% (nasal catheter oxygen inhalation), the low respiratory sound of both the lungs, and no dry and wet rales. The rest of the physical examination is the same as before. Stop vasoactive pressor drugs and start using low-dose β-receptor blockers (metoprolol 12.5 mg/day) that were used to prevent heart failure and VT.

On the tenth day of admission, the temporary pacemaker and gastric tube were removed. Low doses of angiotensin converting enzyme inhibitors (Astat 2 mg) were added to prevent ventricular remodeling. Subsequently, the patient gradually received active physical rehabilitation and comprehensive oral nutrition.

Before discharge, the patient’s body temperature was normal and there was no discomfort such as headache and chest tightness. Re-examination of ECG suggested sinus rhythm and ST-T changes (Figure 1C). Echocardiography indicated that the size of each cardiac cavity was normal, the movement of the ventricular wall was normal, and the ventricular systolic function was normal (LVEF = 59%) (Figures 2D–F). The patient’s chest CT showed little inflammation in the upper left lung (Figure 2I), bilateral pleural effusion, and bilateral lower lung insufficiency (Figure 2L). He was discharged from the hospital on the 21st day of admission and returned home without apparent complications. During the 3-year follow-up period, he had no deterioration of cardiac function, recurrent myocarditis, congestive heart failure, or episodes of ventricular arrhythmias.

During the 21 days of hospitalization, to determine the microbial cause of fever, blood culture, urine culture, stool culture, and fungal D-glucan test were negative and there were no signs of elevated eosinophils and lymphocytes. However, we did not perform an endomyocardial biopsy (EMB) and coronary angiography (CAG) because we failed to obtain the consent of the patient. Due to claustrophobia, the patient failed to undergo a cardiac MR examination. He was diagnosed as an FM case with CGS and MOF.

## Discussion

The precursor symptoms of FM, such as fatigue, fever, cough, dyspnea, and chest pain, are usually not significantly different from the common cold (8). The condition deteriorates rapidly within 2 days to 2 weeks after the onset of prodromal symptoms, namely, severe heart failure, refractory arrhythmia, CGS, and MOF. Hemodynamic disorders need to be treated with vasoactive drugs and MCS devices (6, 9). Studies have shown that in adult patients, FM accounts for about 10% of all the cases of myocarditis, but the mortality rate is more than 50% (10, 11). These characteristics make an early diagnosis, close monitoring, and comprehensive treatment that are very important in the diagnosis and treatment of FM.

The most common causes of FM are viral infections, namely, adenovirus and enterovirus (common CV B serotype) (12–14), cytomegalovirus (15, 16), herpes simplex virus (17), parvovirus B19 (18), H1N1 influenza virus (19), and HIV (20). Due to the lack of specificity and sensitivity, negative serological results cannot exclude possible viral infections. Antiviral therapy is still an important part of FM therapy. Evidence showed that early use of antiviral drugs in patients with viral myocarditis caused by H1N1 could reduce mortality and have a better prognosis (21). Some case reports have shown that antiviral drugs such as oseltamivir and zanamivir had encouraging therapeutic effects (22, 23). The Chinese Society of Cardiology recommended that antiviral treatment can be started as soon as possible for patients with FM (3). In this case, we continued antiviral treatment.

The European Society of Cardiology (ESC) guidelines recommend that the detection of the virus genome by PCR through EMB should be used as the gold standard for the diagnosis of myocarditis (4). EMB could be used to determine specific myocarditis types (such as lymphocytic myocarditis, giant cell myocarditis, and eosinophilic myocarditis) and ruled out cardiac sarcoid, which is of guiding significance for the selection of treatment plan and prognosis of FM (24, 25). A multicenter study showed that only 38% of patients with myocarditis were able to find the viral genome in their EMB samples (26). EMB was at risk of arrhythmia and ventricular perforation due to invasive procedures (27). In this case, due to unstable hemodynamics and lack of available facilities and clinical experience in the early stage of the disease, we decided not to conduct EMB. After the disease was stable, the patient refused to undergo EMB. The ESC myocardial and pericardial disease working group recommends that all the patients with clinically suspected myocarditis should consider CAG. Especially, in the case of CGS, CAG can help guide management strategies (28). In this case, CAG was not performed. The patient had no typical cardiovascular risk factors, no previous history of cardiovascular disease, and extracardiac causes that could explain the symptoms. His left ventricular systolic function and cTnI returned to normal without coronary intervention. In this case, even without available EMB and CAG results, the patient’s clinical manifestations met the diagnostic criteria of FM (ECG/cTnI/echocardiography) (29).

In patients with FM with CGS, appropriate vasopressor and mechanical ventilation can ensure adequate perfusion pressure and oxygenation (7). For patients with FM with poor drug treatment effect, it is recommended to use a temporary MCS support device in time (30). The main purpose of using a temporary MCS support device is to prevent multiple organ dysfunction and death through biventricular unloading, ensure systemic and coronary perfusion and venous dredging, and provide a safe bridge for rehabilitation or durable auxiliary device implantation (31). The most common temporary MCS implantation devices are IABP and venous arterial extracorporeal membrane oxygenator (VA-ECMO) with peripheral cannula (32, 33). IABP inflates and deflates synchronously with systolic and diastolic rhythm through the balloon, which can reduce left ventricular afterload and increase blood flow to the brain and kidney. Compared with the total circulation demand, IABP can provide about 15% additional circulation support. It is the most commercial MCS device for the treatment and the first-line device recommended by some researchers (34, 35). Low-dose inotropic drugs and vasodilators and/or IABP implantation are the most common strategies to reduce cardiac afterload. If IABP cannot sufficiently improve the cycle, VA-ECMO should be considered immediately. VA-ECMO is the preferred temporary MCS option when oxygenation is poor. VA-ECMO can ensure rapid and comprehensive cardiopulmonary assistance. It is an effective method to restore cardiac output and organ perfusion in patients with FM (36). However, VA-ECMO may lead to left ventricular overload and dilation. The increase of left ventricular end-diastolic pressure can reduce coronary blood flow and cause a cardiac electrical storm (37). Therefore, VA-ECMO is best used in combination with IABP, especially for those patients with CGS whose cardiac index is less than 2 l/min/m^2^ or whose blood lactic acid is more than 2 mmol/l. IABP can decrease left ventricular afterload and increase coronary blood flow (38). According to previous reports, the median duration of ECMO was 5–9 days and the therapeutic discharge rate was 55–66% (30, 33, 39). After VA-ECMO implantation, bedside echocardiography was performed everyday to determine whether the ventricular systolic function was restored. Trials of discontinuation could be carried out once there was lasting evidence of cardiac recovery (40, 41). If the cardiac function of patients with FM cannot improve, no matter how many days VA-ECMO supports, durable MCS such as HeartMate II, Heartware HAVAD, and HeartMate 3 LV assist device (VAD) should be considered for treatment. VAD is beneficial to reduce left ventricular load and is a better device to prevent and improve MOF, including liver failure (42, 43). Relevant studies suggested that VAD should be used to replace VA-ECMO when the bilirubin cases rapidly reached more than 3.0 mg/dl (44). In this case, CGS and hypoxia were gradually relieved after using IABP combined with mechanical ventilation and an echocardiographic indication of EF did not decrease further. VA-ECMO was not used.

Whether to use IVIG and steroids in FM is controversial. The position statement of the ESC suggested that immunosuppression therapy should not be started until EMB eliminates viral infection by PCR (4). Studies had shown that early immunosuppression of FM might be the key to hindering the inflammatory process and reduced in-hospital mortality (45, 46). IVIG could eliminate myocardial virus infection faster by targeting specific virus antibodies and reduce cardiac inflammation by regulating the immune response. In the animal model of viral myocarditis, the free immunoglobulin light chain showed antiviral and anti-inflammatory effects, which supported the clinical application of IVIG (47). IVIG could be used for lymphocytic myocarditis in children (48). Several studies have reported that IVIG treatment was effective in the treatment of acute myocarditis and FM (49–51). High-dose intravenous IVIG could significantly improve LVEF and left ventricular end-diastolic diameter (LVEDD) in adult patients with acute myocarditis and FM, reduce the incidence of ventricular tachycardia or fibrillation, and reduce mortality (52–54). Some studies suggested that due to GC-induced immunosuppression, virus infection might worsen and spread and adult lymphocyte FM was a contraindication to immunosuppression (55, 56). A meta-analysis published in 2013 showed that there was no increase in viral replication or disease severity in patients with viral myocarditis treated with GC (57). At the same time, in patients with inflammatory cardiomyopathy and myocarditis with persistent PVB19, the use of immunosuppressive drugs did not aggravate virus replication (58). Studies have shown that the use of GC reduced virus titer by stimulating interferon secretion. In some cases of myocarditis, patients responded well to GC treatment (46, 59). These were evidence of the safety of GC in the treatment of FM. The American Heart Association recently said that if there is a high suspicion of immune-mediated FM, 1 g of methylprednisolone can be taken urgently before biopsy diagnosis or further diagnostic test (7). Ammirati et al. (60) proposed that the combination of immune regulation and MCS using IVIG and GC in FM could help improve circulatory dysfunction and reduce cytokine storm. In this case, we clinically diagnosed the patient as FM, started IVIG and GC treatment for the first time, and the patient responded well to the treatment. It is necessary to carry out large-scale prospective studies to explore the role of immunosuppression in acute and FM, so as to provide evidence for standardized treatment.

In this case, the patient had obvious precursor symptoms of the upper respiratory tract or gastrointestinal virus infection and the symptoms of severe heart failure (rapid deterioration of LVEF and new conduction block) occurred rapidly within 2 weeks of onset. The development of hemodynamic damage was rapid, which required positive inotropic drugs and temporary MCS device implantation support. During hospitalization, his blood bacterial culture and fungal test results were always negative, excluding bacterial or fungal myocarditis. Because eosinophil levels did not rise during hospitalization, he was also less likely to have eosinophilic myocarditis. His left ventricular systolic function and cTnI returned to normal without coronary intervention, which suggests that his myocardial injury was not caused by acute myocardial infarction. Combined with an auxiliary examination (ECG/serum troponin/echocardiography), the patient met the diagnosis of FM. For this FM case, we believe that IABP implantation combined with IVIG, GC, CRRT, and mechanical ventilation is the key to successful treatment.

## Conclusion

For young patients with a history of proinfection and no basis of heart disease, who develop heart failure in a short time, doctors should consider the possibility of FM and improve ECG, echocardiography, and blood biochemical examination as soon as possible. Once FM is suspected, we should be alert to the deterioration of symptoms and hemodynamics caused by acute heart failure or CGS. If there are early signs of circulatory failure, it is recommended to transfer to a tertiary medical center with MCS devices and multidisciplinary teams as soon as possible. The improvement of EMB and CAG has a guiding significance for the treatment and differential diagnosis of FM classification. After the diagnosis of FM, actively use antiviral drugs and drugs to improve circulation. Immunosuppressants can be considered. MCS devices such as IABP are recommended as early as possible before the onset or progression of MOF to maintain stable hemodynamics and perfusion of vital organs. If temporary MCS is invalid, VAD therapy and heart transplantation should be actively considered. Temporary MCS can serve as a bridge for resuscitation or heart transplant transport. The prognosis of patients with FM can be improved by carrying out individualized comprehensive treatment to help patients with FM successfully overcome the acute stage.

## Data availability statement

The original contributions presented in this study are included in the article/Supplementary material, further inquiries can be directed to the corresponding author.

## Ethics statement

Written informed consent was obtained from the participant/s for the publication of this case report. Written informed consent was obtained from the individual(s) for the publication of any potentially identifiable images or data included in this article.

## Author contributions

HL and LL made substantial contributions to conception and design, acquisition of data, or analysis and interpretation of data, took part in drafting the article or revising it critically for important intellectual content, gave final approval of the version to be published, and agreed to be accountable for all the aspects of the manuscript. Both authors contributed to the article and approved the submitted version.

## Conflict of interest

The authors declare that the research was conducted in the absence of any commercial or financial relationships that could be construed as a potential conflict of interest.

## Publisher’s note

All claims expressed in this article are solely those of the authors and do not necessarily represent those of their affiliated organizations, or those of the publisher, the editors and the reviewers. Any product that may be evaluated in this article, or claim that may be made by its manufacturer, is not guaranteed or endorsed by the publisher.
