# Supplementary material for: Successful treatment of fulminant myocarditis with intra-aortic balloon pump counterpulsation combined with immunoglobulin and glucocorticoid in a young male adult
Source: Front Cardiovasc Med. 2022 Jul 22;9:905189. doi: 10.3389/fcvm.2022.905189 (PMC9353579; doi:10.3389/fcvm.2022.905189)
Supplement: Supplementary file 1 [file Table_1.pdf]

Table 1 Laboratory data upon admission

Parameter	Recorded value	Standard value
White blood cell count ($10^{\wedge 9}$/L)	6.75	3.5-9.5
Neutrophils ($10^{\wedge 9}$/L)	4.60	1.8-6.3
Eosinophils ($10^{\wedge 9}$/L)	0.02	0.02-0.52
Lymphocytes ($10^{\wedge 9}$/L)	1.26	1.1-3.2
Hemoglobin (g/l)	133	130-175
Platelet count (($10^{\wedge 9}$/L))	128	128-350
Red blood cell count ($10^{\wedge 12}$/L)	4.32	4.3-5.8
Total bilirubin (umol/l)	27.6	3.4-21.3
Direct bilirubin (umol/l)	13.3	0-6.8
Indirect bilirubin (umol/l)	14.3	1.7-15.4
Alanine aminotransferase (U/L)	21	9-50
Aspartate aminotransferase (U/L)	114	15-40
r -glutamyl transpeptidase (U/L)	18	10-60
Total protein (g/l)	60.2	65-85
Albumin (g/l)	34.9	40-55
Amylase (U/ (g/l))	24	0-103
Creatine (umol/l)	107.5	57-97
Urea nitrogen (mmol/l)	6.97	3.1-8
Uric acid (umol/l)	413	208-428
Serum potassium (mmol/)	3.5	3.5-5.3
Serum sodium (mmol/)	129	137-147
Serum chloride (mmol/)	96	99-110
Serum calcium (mmol/)	1.98	2.11-2.52
Prothrombin time (s)	11.6	9-13
Activated partial thrombin time (s)	38.1	20-40
Thrombin time (s)	17.4	14-21
Fibrinogen (g/l)	4.22	2-4
D-dimer (mg/l)	0.6	0-0.55
N-terminal B-type natriuretic peptide precursor (pg/ml)	8480	0-100
Lactate dehydrogenase (U/L)	406	120-250
Creatine kinase (U/L)	1203	50-310
Creatine kinase isoenzyme (U/L)	55	0-24
serum cardiac troponin I (ng/ml)	38.678	0-0.0026
Procalcitonin (ng/ml)	0.47	0-0.05
Total cholesterol (mmol/l)	2.31	2.8-5.17
Triglyceride (mmol/l)	0.92	0.56-1.70
High density lipoprotein (mmol/l)	0.68	1.2-1.65
Low density lipoprotein (mmol/l)	1.36	2.1-3.1
Free triiodothyronine (pmol/l)	2.87	3.5-6.5
Free tetraiodothyronine (pmol/l)	13.69	11.5-22.7

Serum thyrotropin (pmol/l)	1.03	1.55-4.78
Anti systolic protein antibody	Negative	Negative
Anti-myocardial mitochondrial antibody	Negative	Negative
Anti-myocardial membrane protein antibody	Negative	Negative
Anti-myocardial structural protein antibody	Negative	Negative
Enterovirus RNA	Negative	Negative
Coxsackie virus B_3 IgM antibody	Negative	Negative
Coxsackie virus B_5 IgM antibody	Negative	Negative
Cytomegalovirus	Negative	Negative
HIV virus antibody	Negative	Negative
Syphilis antibody	Negative	Negative
Hepatitis A IgM antibody	Negative	Negative
Anti-HBc antibody	Negative	Negative
Anti-HBs antibody	Negative	Negative
HBs antigen	Negative	Negative
Hepatitis C IgM antibody	Negative	Negative
Hepatitis E IgM antibody	Negative	Negative

IgM: immunoglobulin M.
